# Response of the mesozooplankton community in the western Gulf of Maine to changing oceanographic conditions: the 2010 regime shift

**DOI:** 10.1093/plankt/fbaf066

**Published:** 2026-01-07

**Authors:** Emma C Dullaert, Jeffrey A Runge, Lee Karp-Boss, Shawn Shellito, Cameron R S Thompson, Rebecca J Jones

**Affiliations:** Darling Marine Center, School of Marine Sciences, University of Maine, 193 Clarks Cove Road, Walpole, ME 04573, USA; Darling Marine Center, School of Marine Sciences, University of Maine, 193 Clarks Cove Road, Walpole, ME 04573, USA; School of Marine Sciences and Climate Change Institute, University of Maine, 168 College Ave, Orono, ME 04469, USA; Institute for the Study of Earth, Oceans and Space, University of New Hampshire, 8 College Road, Durham, NH 03824, USA; NERACOOS, 300 Constitution Ave Suite 203, Portsmouth, NH 03801, USA; Darling Marine Center, School of Marine Sciences, University of Maine, 193 Clarks Cove Road, Walpole, ME 04573, USA; North Hampton School, 201 Atlantic Ave., North Hampton, NH 03862, USA

**Keywords:** Calanus finmarchicus, Gulf of Maine, regime shift, mesozooplankton, climate change

## Abstract

The Gulf of Maine has experienced pronounced changes in recent decades, including rapid warming and changes in circulation. Notably, a shift in water masses entering the Gulf occurred around 2010. Concurrent declines in critically endangered North Atlantic right whales, lobster recruitment and abundance of the foundational, subarctic copepod, *Calanus finmarchicus*, have designated the 2010 event as a possible regime shift. We present results from two time series stations documenting change in the mesozooplankton biomass and community composition before and after 2010. We examine both seasonal and interannual variability to elucidate potential changes in phenological drivers of the mesozooplankton population in Wilkinson Basin. Abundances of smaller copepod species increased across all seasons between the two time periods, and significantly lower abundance of late-stage *C. finmarchicus* was observed in late summer through winter, resulting in a decrease in mesozooplankton biomass but increases in biodiversity indices post-2010. The results highlight the contribution of ecologically important increases in chlorophyll-*a* concentration and warmer temperatures as drivers of mesozooplankton growth and reproduction. An important ecological influence on food availability to smaller copepods may be reduced grazing competition by late-stage *C. finmarchicus*, a consequence of its declined abundance due to increased predation loss and reduced advective supply.

## INTRODUCTION

The mesozooplankton community represents an important link in energy transfer from lower to higher trophic levels in the marine environment, both as grazers of primary producers and as a food source for a variety of consumers from pelagic forage fish to baleen whales. Therefore, observing and assessing changes in mesozooplankton diversity and biomass provides a means for understanding broader changes in marine ecosystems and informing management decisions. The Gulf of Maine is a semi-enclosed shelf sea within the broader North Atlantic biome (Pershing and Stamieszkin, 2020). Its mesozooplankton community is largely composed of copepods, with *C. finmarchicus* historically being the dominant species (Bigelow, 1924; Johnson *et al.*, 2011; Melle *et al.*, 2014). Because of its rich lipid stores, *C. finmarchicus* is a valuable energy source for the North Atlantic right whale and many planktivorous pelagic fish such as sand lance and Atlantic herring (Becker *et al.*, 2020; Meyer-Gutbrod *et al.*, 2021; Suca *et al.*, 2022). Other copepods commonly found in the Gulf of Maine, including species in the genera *Centropages*, *Pseudocalanus*, *Metridia* and *Oithona*, are also a food source for planktivores, but have a smaller body size and lipid stores compared to *C. finmarchicus* (e.g. Lee *et al.*, 2006; Carlowicz Lee *et al.*, 2024).

The Gulf of Maine (GoM) mesozooplankton populations exhibit strong seasonal cycles in abundance. For example, *C. finmarchicus* and *Pseudocalanus* spp. reach peak abundance in late spring–early summer, whereas *Centropages typicus* attains peak abundance in late summer–autumn (Durbin and Kane, 2007; Ji *et al.*, 2009; Melle *et al.*, 2014). Seasonal drivers of population dynamics of planktonic copepods in the western GoM include seasonally variable phytoplankton production, advective transport of zooplankton into Wilkinson Basin from the Maine Coastal Current and adjacent deep water in late summer–early autumn (Ji *et al.*, 2022; Runge *et al.*, 2025; Thompson *et al.*, 2025), and predation in Wilkinson Basin during late summer through winter (Wiebe *et al.*, 2022). In addition to seasonal variations, zooplankton populations show interannual and decadal variations in abundance, diversity and evenness (e.g. Pershing *et al.*, 2005; Record *et al.*, 2010, Johnson *et al.*, 2011, Kane, 2007, Pershing and Kemberling, 2023).

In or around 2010, a likely climate-driven shift in the oceanographic regime in the Gulf of Maine occurred, characterized by a marked increase in sea surface temperature (GMRI, 2022) and a change in the external sources of water (Record *et al.*, 2019; Meyer-Gutbrod *et al.*, 2021; Townsend *et al.*, 2023). Importantly, an increased influence of Warm Slope Water driven by a northward shift in the Gulf Stream is considered to have supplanted the flow of Labrador Slope Water into the deep Gulf of Maine (Thibodeau *et al.*, 2018, Seidov *et al.*, 2021; Gonçalves Neto *et al*., 2021). The inflow of Scotian Shelf Water during winter through spring, which sets up a seasonal barrier to intrusions of Warm Slope Water and modified Gulf Stream water later in the year, is also variable (Townsend *et al.*, 2023). These water masses contain different populations of mesozooplankton and therefore influence the abundance and composition of zooplankton that enter into the Gulf of Maine (Johnson *et al*., 2011; Record *et al.*, 2019; Runge *et al.*, 2025).

Concurrently with the 2010 shift in oceanographic conditions, marked changes have been observed in zooplankton populations. These include a decline in abundance of summer through winter late-stage *C. finmarchicus* (Record *et al.*, 2019; Meyer-Gutbrod *et al.*, 2021; Pershing and Kemberling, 2023; Shank *et al.*, 2024; Runge *et al.*, 2025), an increase in the abundance of early-stage *C. finmarchicus* copepodid stages in spring and early summer (Ji *et al.*, 2022; Honda *et al.*, 2024; Runge *et al.*, 2025), and an increase in the abundance of smaller copepods, including *Centropages*, *Pseudocalanus* and *Metridia* species (Pershing and Kemberling, 2023). These changes in the mesozooplankton community drew interest because of their potential link to declines in the populations of endangered and commercially important species (Record *et al.*, 2019; Meyer-Gutbrod *et al.*, 2021; Richards and Hunter, 2021). Notably, the foraging behavior of the critically endangered North Atlantic right whale population has shifted in the past decade, moving from their traditional summer feeding grounds in the eastern Gulf of Maine and Bay of Fundy to the Gulf of St. Lawrence (Meyer-Gutbrod *et al.*, 2021). Recent declining trends in lobster recruitment have also been linked to *C. finmarchicus* abundance and phenology (Carloni *et al.*, 2024; Shank *et al.*, 2024). These oceanographic and ecosystem changes occurring in the Gulf of Maine around 2010 have been labeled as a regime shift in a number of studies (Meyer-Gutbrod *et al.*, 2021; Pershing and Kemberling, 2023; Shank *et al.*, 2024).

To explain the shift in the mesozooplankton community in 2010, Pershing and Kemberling (2023) proposed that decadal variations in community structure are driven by external physical forcings that enhance water column stratification in the Gulf of Maine favoring “summer-time communities” of the smaller mesozooplankton taxa (e.g. *Centropages*, *Pseudocalanus*, *Metridia* and *Oithona*). In addition, there is evidence for increased invertebrate predation resulting in lower autumn–winter abundance of *C. finmarchicus*, which could alter the mesozooplankton community structure given the role of *C. finmarchicus* as competitive grazers and predators on early life stages of other copepods (Ji *et al.*, 2022; Wiebe *et al.*, 2022; Pershing and Kemberling, 2023; Honda *et al.*, 2024, 2025).

The effects of the 2010 shift in oceanographic conditions and the mechanisms underlying change in the Gulf of Maine mesozooplankton community merit further investigation, not only to better understand how the western Gulf of Maine ecosystem will respond to future oceanographic change, but also to provide insight into mechanisms driving mesozooplankton dynamics across the margin of the North Atlantic biome. Here, we use data from two time series stations in the western Gulf of Maine to document change in the zooplankton community in the periods immediately before and after the 2010 oceanographic shift. We apply the lens of seasonality to examine how abundances of *C. finmarchicus* and other species, and the overall diversity of the mesozooplankton community, responded at subannual to annual scales and whether changes in abundance and composition affected total mesozooplankton biomass. Using available *in situ* data of chlorophyll-*a* concentrations and temperature from these two time series stations, as well as parameters and calculations from literature reviews, we evaluate the relative role of temperature versus food on potential changes in copepod growth, fecundity and mortality. Observations from these two time series stations provide insight into the complex interactions shaping the mesozooplankton community in the western Gulf of Maine, particularly the roles of external advective supply, temperature, local production and trophic interactions. Understanding these drivers and their mechanistic linkages to lower trophic levels provides a more complete view of ecosystem dynamics in a region that has undergone regime shifts in the past and is expected to continue experiencing them, with especially significant socioeconomic consequences.

## MATERIALS AND METHODS

### Sampling locations

**Fig. 1:**
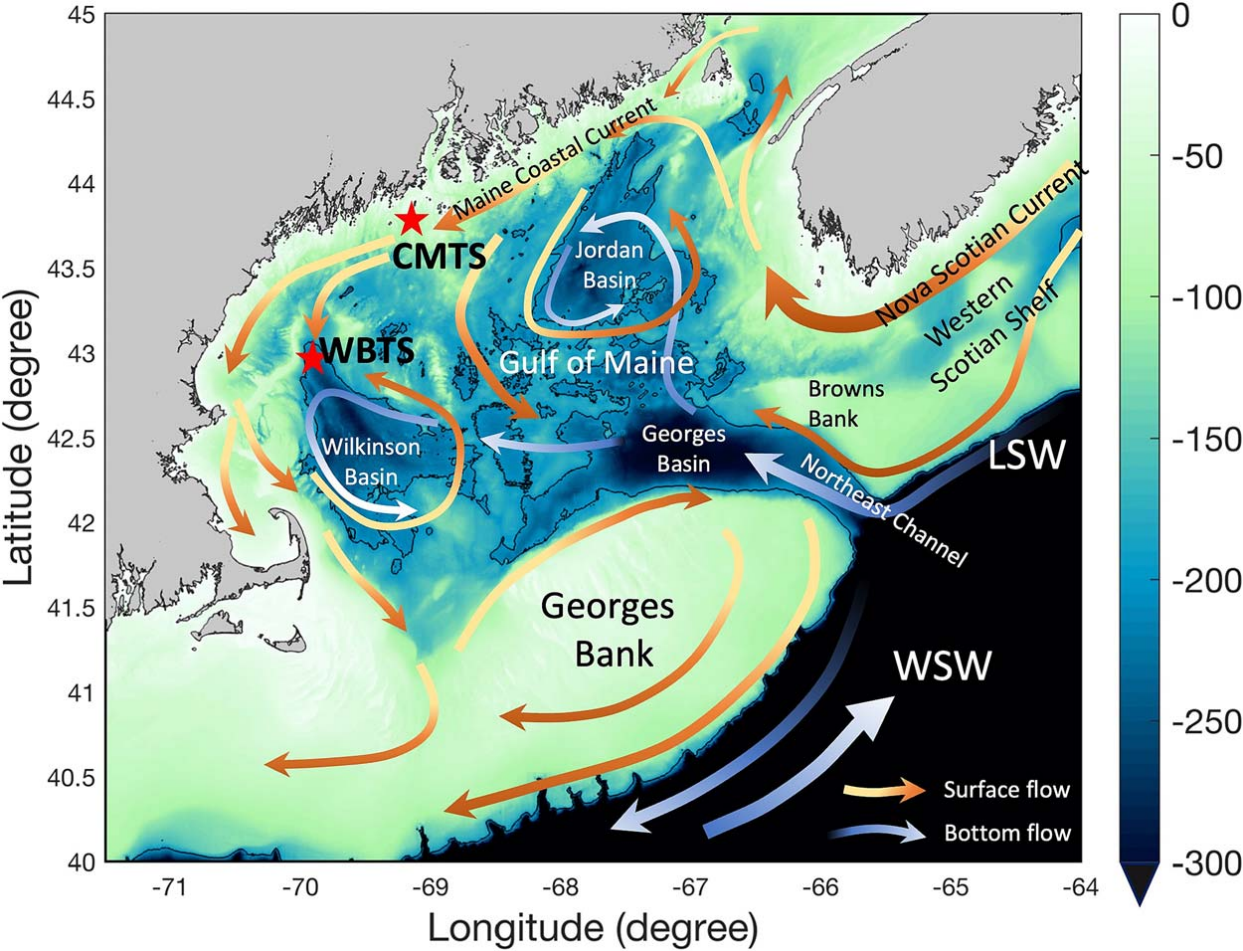
The Gulf of Maine, showing the location of the WBTS and CMTS stations as well as the general circulation pattern of surface and bottom flows and bathymetric features (color scale in m beside right axis). LSW indicates the general location at the shelf break of Labrador Slope Water derived from the Labrador Current; WSW indicates general location of Warm Slope Water west of the Gulf Stream. Figure courtesy of Rubao Ji.

Zooplankton samples were collected at two long-term time series stations in the Gulf of Maine (Fig. 1), now maintained by the Northeastern Regional Association of Coastal Ocean Observing Systems (NERACOOS) as part of the U.S. MBON (Marine Biodiversity Observation Network). The Wilkinson Basin Time Series station (WBTS: 42.914° N, 69.786° W; bottom depth 257 m) is located ~38 nautical miles offshore in the northwest corner of Wilkinson Basin the primary deep basin in the western GoM harboring high abundances of diapausing stage CV of *C. finmarchicus* and serves as a primary source of *C. finmarchicus* to fisheries and migrating right whales (Lynch *et al.*, 1998; Miller *et al.*, 1998; Thompson *et al.*, 2025). The Coastal Maine Time Series (CMTS) station (43.747° N, 69.502° W; bottom depth: 105 m) is located along the coastal edge of the Maine Coastal Current, a current system that likely plays an important role in maintaining populations of mesozooplankton in the western GoM by serving as both a source of elevated summer food availability and supply of individuals from the eastern GoM (Runge *et al.*, 2015; Ji *et al.*, 2017). Note that zooplankton sampling at CMTS started in 2008, so there are only 2 years of data available before the shift in oceanographic conditions observed in 2010.

At the WBTS station, monthly samples of zooplankton and environmental variables (temperature, salinity, chlorophyll-*a*) were collected, with some gaps, for a total of 38 times between January 2005 and August 2008 from the R/V *Gulf Challenger*, operating out of the University of New Hampshire’s Judd-Gregg Marine Research Complex in Newcastle, New Hampshire. Due to funding constraints, the station was not visited in 2009 or 2011 and only once in 2010. It was then sampled at approximately monthly intervals, with gaps, for a total of 43 times between April 2012 and July 2017. At the CMTS station, samples were collected from the R/V *Ira C*, operating out of the University of Maine’s Darling Marine Center in Walpole, Maine. The CMTS station was first sampled once in April and in September 2007, then primarily in spring through early autumn at approximately monthly intervals for a total of 134 times through July 2017. The temporal distribution of sampling at these two stations over the duration of the study can be seen in the supplementary time series plots (Figs. S1–S3) and is available in the archived data files (see Data Archiving).

### Mesozooplankton biomass, abundance and community composition

Mesozooplankton samples were collected with a 200-μm-mesh ring net. Following Canadian Atlantic Zone Monitoring Program (AZMP) protocols (Mitchell *et al.*, 2002), vertical tows from 5 to 7 m above the bottom to the surface were conducted at a rate of 40 m/min, using a 0.75-m-diameter single-ring net at CMTS and a SEA-GEAR Model 9600 0.75-m-diameter twin-ring net at WBTS. Two vertical casts were performed at each site and sampling day. The contents of the cod ends were concentrated and poured into 500-mL sample jars containing 50 mL of 40% buffered formaldehyde and topped off with seawater as needed, resulting in a final formaldehyde concentration of 4%. In the laboratory, samples were split in half using a Folsom splitter under a fume hood, one marked for biomass and one for species composition analysis. The biomass subsamples were filtered on two pre-weighed filters cut from the 200-μm or finer Nitex mesh. The filtered samples were rinsed with 100 mL of freshwater to remove residual salt water and placed in a drying oven at 65°C for 24–48 h. Dried samples were removed from the oven and weighed immediately on a Mettler Toledo PG403-S DeltaRange microbalance. Sample dry weight (DW) was obtained by subtracting the initial mass of the filters from the final mass. Biomass (g/m^2^) was calculated as follows:


$$ Volume\ filtered\ by\ the\ net= net\ depth\ast net\ area $$

$$ Biomass\ \left(\frac{g}{m^2}\right)=\left(\frac{sample\ DW}{volume\ filtered\ by\ the\ net}\right)\ast net\ depth $$

For zooplankton abundance and community composition analysis, the half sample from one of the net tows was drained of formaldehyde solution using a 200-μm-mesh sieve. The rinsed samples were then diluted in a known volume of filtered seawater depending on the density of the sample. Aliquots of 5 mL were then taken with the nominal goal to identify and enumerate a minimum of 50 *C. finmarchicus* and a minimum of 200 mesozooplankton generally. Macrozooplankton (e.g. Ctenophora, Chaetognatha and Cnidaria) were counted from the whole half sample. Multiple aliquots were frequently taken to achieve those goals. Where *C. finmarchicus* had relatively low abundance compared to other species, additional aliquots were taken for their identification alone. On average 390 animals (copepodid stages or individuals of other taxa) were identified from each sample with a maximum of just >1000 identified from a single sample. Usually, the half samples from the other net tow were analyzed for biomass and, on occasion, also the *C. finmarchicus* abundance, but not for other zooplankton abundance. In these cases, the mean biomass and *C. finmarchicus* abundance were used. Species and taxon counts were conducted under a Leica MZ6 Modular Stereomicroscope. Abundances (individuals/m^2^) were calculated as follows:


\begin{align*}& Dilution\ factor=\frac{dilution}{aliquot} \\& Species\ or\ taxon\ abundance\ \left(\frac{individuals}{m^2}\right)\\&=\frac{\# of\ individuals\ast dilution\ factor\ast split}{volume\ filtered\ by\ the\ net}\ast net\ depth \end{align*}

### Environmental variables

At the WBTS station, environmental data were acquired on each cruise using dedicated water sampling systems. Vertical profiles of temperature and salinity were measured from 5 m above the bottom to the surface with a Sea-Bird Electronics (SBE) 25Plus CTD. From these profiles, potential densities were calculated, and winter mixed layer depths were estimated following Cai *et al.* (2021). Vertical changes in potential density, at 1-m-depth intervals relative to a reference value at 3 m, were calculated until the difference exceeded a threshold value of 0.04 kg/m^3^ for at least 5 consecutive depths. We chose the threshold value from the literature that best represented our visual inspection of the mixed layer in density profiles at the WBTS station. The identified threshold depth was then used to represent the mixed layer depth of each profile.

Vertical distributions of chlorophyll-*a* concentration at the WBTS station were estimated from chlorophyll fluorescence, measured with a Wetlabs *in situ* fluorometer (Wetlabs Eco FL3 mounted on an optical profiler prior to 2014 and a Wetlabs Wetstar mounted on the SBE-55 CTD rosette thereafter). Water samples were collected at 3–4 depths (2, 10, 20 and 40 m through 2010; 2 m, depth of chlorophyll maximum and 50 m thereafter) during each profile for the calibration of the *in situ* fluorometer. Samples were filtered onboard the vessel using glass fiber filters (GF/F) and stored in liquid N until further processing on shore. Laboratory extraction and calculations of chlorophyll-*a* concentration followed the method described in Strickland and Parsons, 1972.

CTD and chlorophyll data at the CMTS station were not processed routinely, especially during the first several years of the time series and are therefore not included here.

### Data analysis

Given the role that seasonality plays in the mesozooplankton, long-term trends could be masked by seasonal variability. Therefore, zooplankton dry weights and mean abundances of mesozooplankton were divided into three phenological seasons based on copepod life history markers, spring (Apr–Jul; spring production), summer (Aug–Oct; summer–autumn growth) and winter (Nov–Mar; winter dormancy). Throughout the manuscript, any mention of season will refer to phenological seasons as defined here, rather than traditional meteorological seasons. Trends were assessed separately for each season. This grouping differs somewhat from the quarterly seasons reported by Runge *et al.* (2025) but provides sufficient data in each period for statistical analysis.

Zooplankton biomass and integrated chlorophyll-*a* standing stock were plotted according to season. The percent contribution of *C. finmarchicus* to total mesozooplankton biomass was estimated for each sample by multiplying the number of individuals by average dry weight estimates of *C. finmarchicus* (Table 1; Head and Harris, 2004; C. Johnson personal communication) for each life stage (copepodid stages CI–CV, male, female) and dividing by the total sample dry weight. Trends in the data for biomass, estimated percent *C. finmarchicus* contribution and chlorophyll-*a* standing stock were determined by both linear regression and a non-parametric (Wilcoxon rank sum test) comparison of values for the periods before and after 2010; significance of the trends was determined based on a *P*-value < 0.05. We primarily present the pre-/post-2010 analysis here, but all regressions can be found in the supplementary  materials.

**Table 1:** *Estimated dry weight values (μg) for each life stage (copepodid stages CI–CV*, *male*, *female) of Calanus finmarchicus in the Gulf of Maine (adapted from*  Head and Harris, 2004*; C. Johnson personal communication)*

CI	CII	CIII	CIV	CV	CVI-M	CVI-F
3.1	8.4	24.4	87.3	236.2	249.7	323.4

Abundance data (as ind. m^−2^) for individual taxa were averaged over the designated periods before and after the proposed regime shift in 2010 both for the whole year (referred to as annual) and for each season separately. Median abundances for each time period were ranked, and the 16 most abundant copepod taxa were used for the visualization of the data. The abundance value for each taxon represents the estimated abundance of copepodid and adult life stages. The non-parametric Wilcoxon rank sum test was used to determine whether differences in abundance of each taxon before and after 2010 were significant. To assess the structure of the entire mesozooplankton community, all abundance data were organized into 11 major taxonomic groups (Copepoda, Other Crustacea, Urochordata, Ctenophora, Annelida, Mollusca, Bryozoa, Protista, Chaetognatha, Cnidaria, miscellaneous). Abundances of the species within each of these taxa were calculated and compared before and after 2010, and significant differences were discerned using the Wilcoxon rank sum test.

To assess potential changes in biodiversity, the Shannon–Wiener index and Pielou *J* evenness index (where *p* is the proportion of each species to the entire community, *S* is the number of species in the community):

**Shannon–Wiener diversity index**


$$ {H}^{\prime }=-{\sum}_{i=1}^S{p}_i\ \ln\{p}_i $$

**Pielou *J* evenness index**


$$ {J}^{\prime }=\frac{H^{\prime }}{\ln (S)} $$

were calculated for each sample and averaged over each season for the periods before and after 2010 to identify potential changes in biodiversity; significance was determined using a Wilcoxon rank sum test. Change in average species richness (# of species present in each sample) is also reported.

The average temperature and salinity at the WBTS station in the estimated mixed layer, 0–50 m layer and two deep layers (100–125 m and 200–225 m) bracketing the overwintering habitat of *C. finmarchicus* were calculated for each sampling date. These values were then averaged over the years before and after 2010 on an annual scale and for each season separately. Data from the two time periods were compared using a non-parametric Wilcoxon rank sum test at a 95% confidence level.

## RESULTS

### Mesozooplankton biomass

**Fig. 2:**
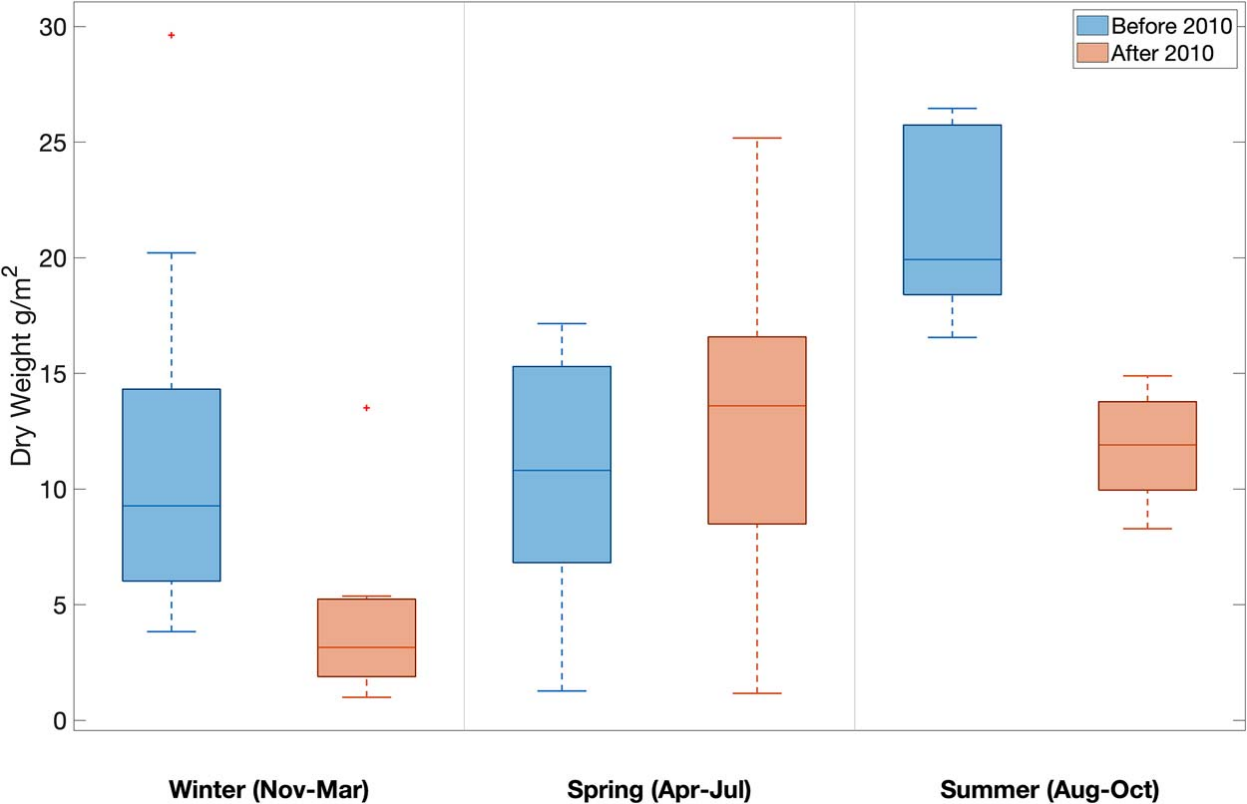
Mesozooplankton dry weight (g/m^2^) before (2005–2010; left) and after (2011–2017; right) the 2010 change in oceanographic conditions in the Gulf of Maine at the Wilkinson Basin Time Series station (WBTS). The median is represented by a horizontal line through the box. The top and bottom lines represent the 75th and 25th percentiles, respectively. The whiskers represent the minimum and maximum values, and any outliers are indicated with a plus (+) symbol. Decreases were significant in both winter (*P* = 0.006) and summer (*P* = 0.001), and there was no significant change in the spring (*P* = 0.314), as determined by Wilcoxon rank sum test.

**Fig. 3:**
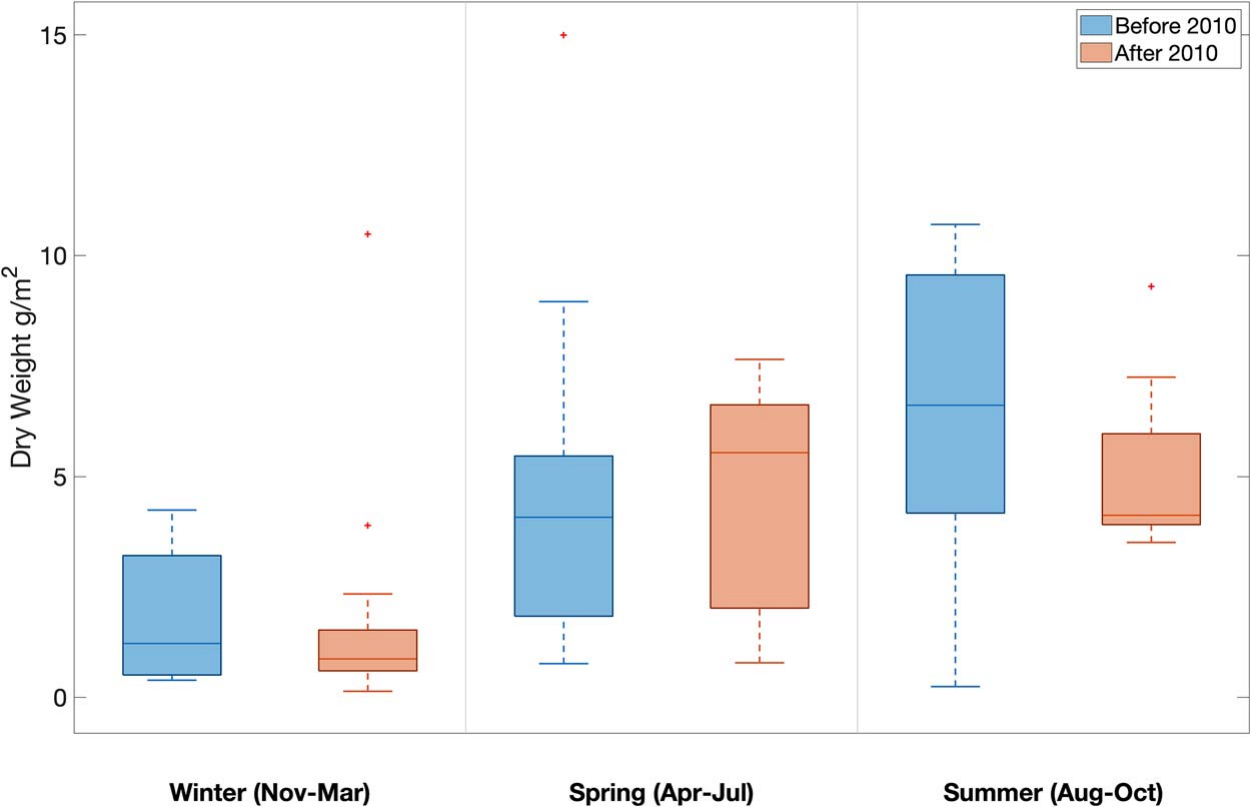
Mesozooplankton dry weight (g/m^2^) before (2005–2010; left) and after (2011–2017; right) the 2010 change in oceanographic conditions in the Gulf of Maine at the Coastal Maine Time Series station (CMTS). The median is represented by a horizontal line through the box. The top and bottom lines represent the 75th and 25th percentiles, respectively. The whiskers represent the minimum and maximum values, and any outliers are indicated with a plus (+) symbol. No significant changes were found in winter (*P* = 0.510), spring (*P* = 0.489) or summer (*P* = 0.113) as determined by Wilcoxon rank sum test.

Relative changes in mesozooplankton biomass (measured as dry weight) between the two periods (pre- and post-2010) at the WBTS station varied between seasons. Mesozooplankton biomass was significantly lower in the period after 2010 in the winter (a 66% decline; *P =* 0.006) and the summer (a 67% decline; *P* = 0.001) and showed no significant change in the spring (*P* = 0.314, Fig. 2). There was also a phenological change. Before 2010, mesozooplankton biomass was highest in the summer and lowest in the spring (Fig. 2). Median dry mass was 9.3 g/m^2^ in the winter, 10.8 g/m^2^ in the spring and 19.9 g/m^2^ in the summer (Fig. 2). After 2010, mesozooplankton biomass at WBTS was highest in the spring and lowest in the winter (Fig. 2). Median dry weight was 3.2 g/m^2^ in the winter, 13.6 g/m^2^ in the spring and 11.9 g/m^2^ in the summer (Fig. 2). At the CMTS station, there was no change in biomass during the period 2008–2017 (Fig. 3). Median dry mass before 2010 was 1.2 g/m^2^ in the winter, 4.1 g/m^2^ in the spring and 6.6 g/m^2^ in the summer, and 0.9 g/m^2^, 5.5 g/m^2^ and 4.1 g/m^2^ after 2010, respectively (Fig. 3).

**Fig. 4:**
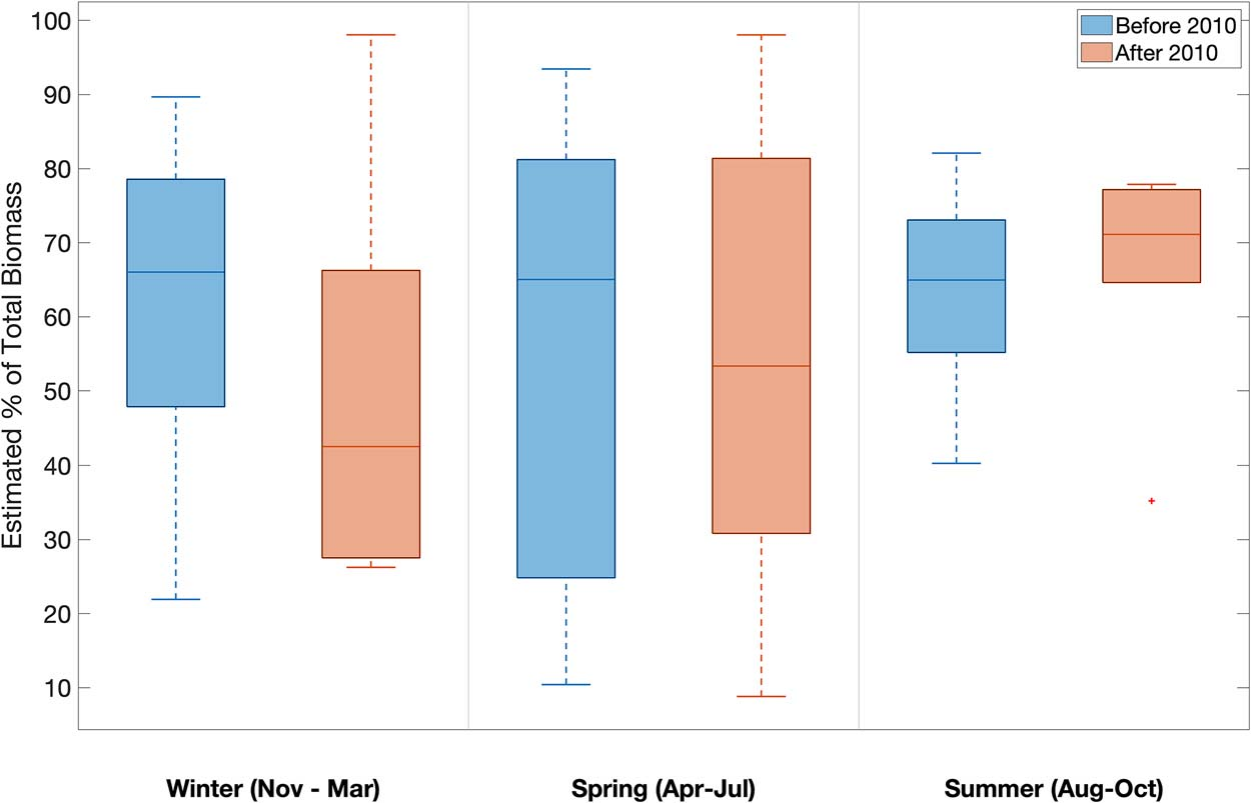
Estimated contribution (%) of *Calanus finmarchicus* (stages CI–CV) biomass, as determined by published dry weight values, to the total zooplankton biomass at the Wilkinson Basin Time Series station (WBTS) before (2005–2010; left) and after (2011–2017; right). The median is represented by a horizontal line through the box. The top and bottom lines represent the 75th and 25th percentiles, respectively. The whiskers represent the minimum and maximum values, and any outliers are indicated with a plus (+) symbol. No significant changes were found in winter (*P* = 0.315), spring (*P* = 1.000) or summer (*P* = 0.573) as determined by Wilcoxon rank sum test.

The relative contribution of *C. finmarchicus* to total zooplankton biomass at the WBTS station also varies between the examined period and between seasons (Fig. 4). As calculated from observed abundance and the dry weight values for each life stage (Table 1), before 2010 the median estimated *C. finmarchicus* biomass at WBTS constituted 64% of the total mesozooplankton biomass in winter and 65% in both spring and summer. After 2010, *C. finmarchicus* accounted for 47% in the winter, 53% in spring and 71% in summer (Fig. 4). However, none of the changes in estimated percent contribution of *C. finmarchicus* to the total mesozooplankton biomass were statistically significant, indicating that any increases in abundance of smaller copepod species did not offset the biomass decline due to loss in abundance of the much larger *Calanus.*

### Mesozooplankton community composition and abundance

**Fig. 5:**
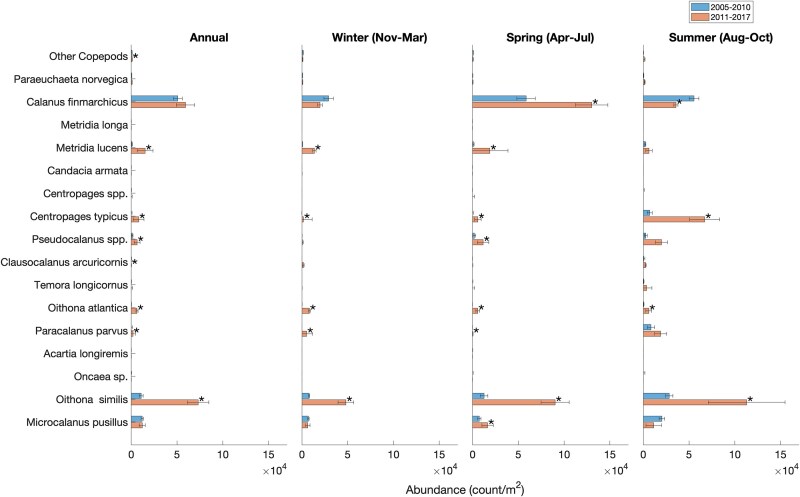
Median abundance (individuals/m^2^) of copepod species before (2005–2010; top) and after (2011–2017; bottom) the 2010 oceanographic regime shift in the Gulf of Maine at the Wilkinson Basin Time Series station (WBTS). Statistically significant changes are indicated with a star symbol (*). Species are ordered by size with the smallest at the bottom and the largest at the top as determined by their prosome length.

**Fig. 6:**
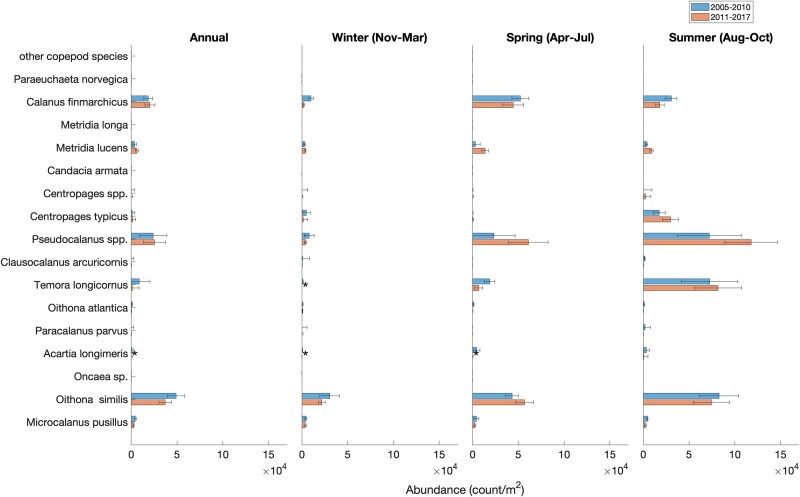
Median abundance (individuals/m^2^) of copepod species before (2005–2010; top) and after (2011–2017; bottom) the 2010 oceanographic regime shift in the Gulf of Maine at the Coastal Maine Time Series station (CMTS). Statistically significant changes are indicated with a star symbol (*). Species are ordered by size with the smallest at the bottom and the largest at the top as determined by their prosome length.

**Fig. 7:**
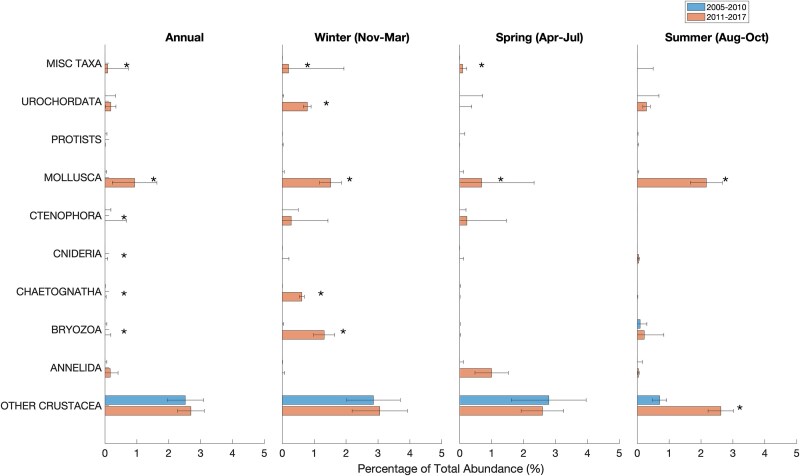
Mean percentage of non-copepod taxonomic groups to the total mesozooplankton abundance before (2005–2010; top) and after (2011–2017; bottom) the 2010 oceanographic regime shift in the Gulf of Maine at the Wilkinson Basin Time Series station (WBTS). Statistically significant changes are indicated with a star symbol (*).

**Fig. 8:**
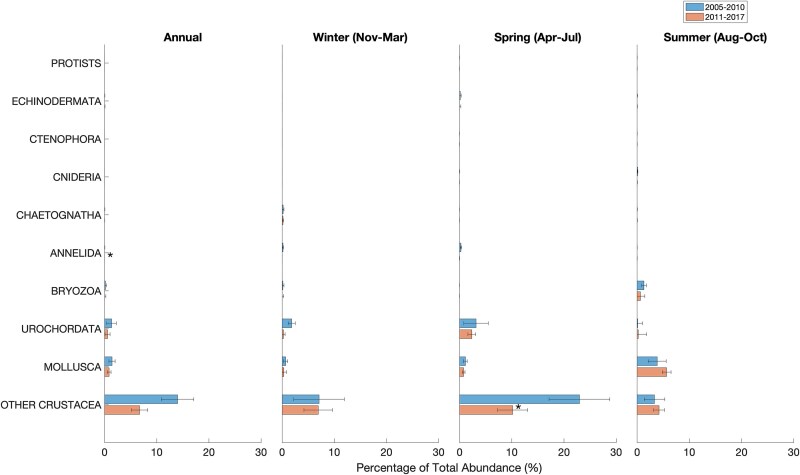
Median percentage of non-copepod taxonomic groups proportion of mesozooplankton taxa to the total mesozooplankton abundance before (2005–2010; top) and after (2011–2017; bottom) the 2010 oceanographic regime shift in the Gulf of Maine at the Coastal Maine Time Series station (CMTS). Statistically significant changes are indicated with a star symbol (*).

Across all years, the same 16 taxa (at the species or genus level) of planktonic copepods constituted the majority of the mesozooplankton community and there was no indication of introduction of new species during the period of study in both sites (Figs 5 and 6). The most prominent of copepods were *C. finmarchicus*, *Pseudocalanus* spp., *Oithona* spp., *Metridia* spp., *Microcalanus* spp. and *Centropages* spp. (Figs 5 and 6). Collectively, other major taxonomic groups (Figs 7 and 8) accounted for less than a quarter of the total mesozooplankton abundance at both stations (4–13% at WBTS and 15–25% at CMTS). Averaged over the entire year, the median proportion of copepod abundance to total mesozooplankton abundance at the WBTS station decreased significantly from 96% before 2010 to 87% after 2010 (Wilcoxon rank sum test; *P* < 0.01); there was no significant change in the proportion of copepod abundance at CMTS (*P* = 0.09). The contribution to the total mesozooplankton of most other major taxa at WBTS, including ctenophores, mollusks, bryozoans, chaetognaths and cnidaria, increased significantly (Fig. 7). There was also a significant increase in the “miscellaneous taxa” that mainly consists of trochophores, which could be larvae of either annelids or mollusks (Fig. 7).

Before 2010, the copepod assemblage at the WBTS station was dominated by *C. finmarchicus*. The next most abundant copepod taxa were *Oithona* (predominantly *O. similis*) and *Microcalanus* (assumed to be *M. pusillus*). After 2010, *Oithona similis* was the most abundant species. Abundances of smaller copepod species increased significantly, most notably in species of *Centropages* (mostly *C. typicus*), *Pseudocalanus* and *Metridia* (mostly *M. lucens* but also *M. longa*) (Fig. 5). The annual Shannon–Wiener biodiversity index increased during the period of study from 1.53 to 1.89, driven by an increase in species evenness and richness, from 0.60 before 2010 to 0.65 post-2010, and 14 to 17 species, respectively (*P* < 0.01; Table 2).

**Table 2:** *Median Shannon–Wiener index (H)*, *Pielou’s (J) evenness index and species richness (R*, *# of taxa) for the mesozooplankton community before (2005–2010) and after (2011–2017) the 2010 oceanographic shift in the Gulf of Maine at the Wilkinson Basin Time Series station (WBTS)*

Season	Period	Shannon–Wiener index (*H*)	Pielou’s evenness index (*J*)	Richness (*S*)
**Annual**	2005–2010	**1.53**	**0.60**	**14**
	2010–2017	**1.89**	**0.65**	**17**
**Winter**	2005–2010	**1.32**	**0.63**	**10**
	2010–2017	**2.05**	**0.71**	**18**
**Spring**	2005–2010	**1.46**	**0.55**	14
	2010–2017	**1.66**	**0.63**	16
**Summer**	2005–2010	**1.72**	0.63	**15**
	2010–2017	**2.01**	0.70	**19**

Pairs with statistically significant changes (Wilcoxon rank sum test: *P* < 0.05) are bolded

Averaged over an annual scale, there was no significant change observed in the abundance of *C. finmarchicus* stages CI–CVI at the WBTS station before and after 2010 (Fig. 5). However, since the primary drivers of zooplankton abundance in the western Gulf of Maine are seasonally variable, averaging over an annual scale may obscure subannual changes in the zooplankton community (Ji *et al.*, 2010; Runge *et al.*, 2025). During the spring phenological period, *C. finmarchicus* abundance increased significantly immediately after 2010, whereas abundance in summer declined significantly (Fig. 5). Although a negative trend in *C. finmarchicus* abundance was also observed in the winter at the WBTS station, the change was not statistically significant.

A significant increase in the abundances of many smaller copepod species was observed post-2010 across all seasons (Fig. 5). The abundance of *O. similis* increased by a factor 4 in the spring and a factor of 8 in the summer and winter. *Oithona atlantica* and *C. typicus* also saw significant increases across all seasons. The abundance of *Pseudocalanus* spp. and *Metridia lucens* increased, particularly in spring, whereas *Paracalanus* spp. (assumed to be *P. parvus;* juveniles may include some *Parvocalanus* and *Clausocalanus*) exhibited significant increases in the autumn, and *C. typicus* increased significantly in all seasons, particularly in summer (Fig. 5). Between the pre- and post-2010 periods, median Shannon–Wiener diversity increased significantly in the winter (from 1.32 to 2.05; *P* ≤ 0.01), spring (1.46 to 1.66; *P* = 0.02) and summer (from 1.72 to 2.01; *P* = 0.01), species evenness increased significantly across all seasons except summer (*P* = 0.23) and richness increased significantly in all but the spring (*P* = 0.07) (Table 2). The observed increases in richness are the result of more frequent occurrence of previously less common species in the samples, especially during the winter, as is evident in Fig. 5. Therefore, on any given sampling date, a less common species would show up, increasing richness and the overall median for the latter time period. The changes in the Shannon–Wiener indices, which reflect changes in both species’ evenness and species richness, indicate an overall more balanced mesozooplankton community with less dominance of *C. finmarchicus* and an increased presence of smaller copepods in Wilkinson Basin post-2010.

At the CMTS station, copepods made up 72% of the total zooplankton abundance before and 81% after 2010. The abundance of annelids decreased significantly, but no significant changes were observed in any of the other taxonomic groups (Fig. 8). At the CMTS station, abundances of copepod species remained fairly constant during the period 2010–2017 (Fig. 6). In general, the annual mean total mesozooplankton abundance was on the same order of magnitude at both stations and most species are present at both. However, relative proportions varied. The relative abundances of *C. finmarchicus* and *M. lucens* were about twice as high at the deeper WBTS station, as compared to the CMTS station, whereas the relative abundances of *Pseudocalanus* and *Temora* were notably higher at the CMTS station (Figs 5 and 6).

### Environmental variables

We observed increases in temperature at the Wilkinson Basin time series station across all seasons and depths between the periods pre- and post-2010. The annual median temperature in the 0–50-m, 100–125-m and 200–225-m layers at the Wilkinson Basin station increased significantly, by 1.3–3°C, post-2010 (Table 3). The annual median salinity increased significantly in the 0–50- and 100–125-m layers (Table 3). The changes in temperature and salinity were reflected in winter, spring and summer seasons. Summer median temperatures in the 0–50-m layer increased by 2.8°C and 1.9°C and 1.3°C, respectively, in the 100–125-m and 200–225-m layers, respectively. In winter, the surface layer temperatures increased by 2°C and temperatures in the deep layers increased by 1.3°C. In spring, surface layer temperatures rose 1.6–1.8°C in the mixed layer and between 0 and 50 m (Table 3). On average, the mixed layer is deepest in the winter in the period before 2010 (Table 3). No data for November were available before 2012, but removing post-2010 November data from the analysis did not alter the results. The seasonally averaged mixed layer depth was not significantly different before and after 2010 in any season (Table 3).

**Table 3:** *Median values of environmental variables temperature*, *salinity*, *mixed layer depth and chlorophyll-a concentration (averaged 0–50 m) before (2005–2010) and after (2011–2017) the 2010 oceanographic shift in the Gulf of Maine at the Wilkinson Basin Time Series station (WBTS)*

	Temperature (°C)							
	Mixed layer		0–50 m		100–125 m		200–225 m	
	**2005–2010**	**2011–2017**	**2005–2010**	**2011–2017**	**2005–2010**	**2011–2017**	**2005–2010**	**2011–2017**
Annual	8.7	11.7	**5.9**	**7.8**	**5.1**	**6.4**	**6.8**	**8.1**
Winter	**5.2**	**7.2**	**5.2**	**7.2**	**5.9**	**7.2**	**6.8**	**8.2**
Spring	8.9	13.1	**5.8**	**7.5**	**4.7**	**6.11**	**6.8**	**7.5**
Summer	16.9	16.9	**9.9**	**12.7**	**5.4**	**7.3**	**7.0**	**8.3**
	**Salinity (psu)**							
Annual	32.3	32.5	**32.4**	**32.7**	**33.1**	**33.2**	34.0	34.1
Winter	**32.8**	**33.1**	**32.8**	**33.1**	33.2	33.3	34.1	34.2
Spring	32.1	32.0	32.3	32.4	**33.0**	**33.1**	34.0	33.8
Summer	31.9	32.0	32.4	32.6	**33.0**	**33.1**	34.0	34.1
	**Mixed layer depth (m)**							
Winter	84	77						
Spring	9	7.5						
Summer	9	10.3						

Pairs with statistically significant (Wilcoxon rank sum test: *P* < 0.05) changes are bolded

**Fig. 9:**
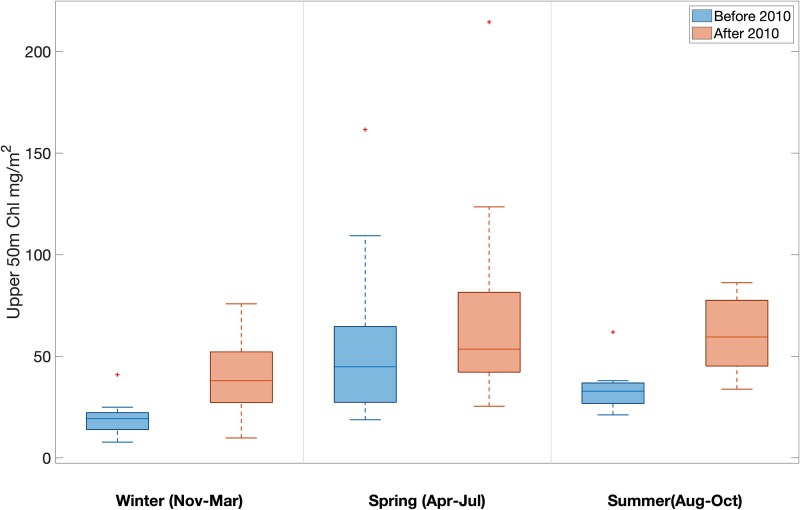
Median integrated chlorophyll-*a* standing stock (mg Chl *a* m^−2^) in the upper 50 m of the water column at the Wilkinson Basin Time Series station during the winter, spring and summer in each time period. Chlorophyll-*a* standing stock significantly increased in the winter (from 19.5 to 38 mg Chl *a* m^−2^: *P* = 0.001) and summer (from 33 to 60 mg Chl *a* m^−2^: *P* = 0.003) and no significant trend in the spring (*P* = 0.114).

In general, chlorophyll-*a* concentrations at WBTS were highest in the spring/early summer and lowest in the late autumn/winter period. Before the shift in oceanographic conditions in 2010, median integrated 0–50 m chlorophyll-*a* standing stock was 19.3 mg/m^2^ in winter, 44.9 mg/m^2^ in spring and 32.8 mg/m^2^ in summer (Fig. 9). In the period after 2010, standing stocks were significantly higher in winter (38 mg/m^2^; *P =* 0.001) and in summer (59.5 mg/m^2^; *P =* 0.003), but not in the spring (53.5 mg/m^2^; *P =* 0.114) (Fig. 9). The increases in chlorophyll standing stock suggests an increase in phytoplankton biomass at WBTS.

## DISCUSSION

We focus here on change in Wilkinson Basin, the primary deep basin in the western Gulf of Maine. The WBTS station is representative of the abundance of *C. finmarchicus* more broadly across Wilkinson Basin (Runge *et al.*, 2015, 2025), and we infer that this representation also applies to the mesozooplankton community generally. Because sampling at the CMTS station started only in 2008, with therefore fewer data before 2010, any conclusions about changes in the mesozooplankton community along the Maine coast would be uncertain.

The prominent changes at the WBTS station include a decline in the abundance of *C. finmarchicus* in summer/early autumn, an increase in abundance of other copepod and mesozooplankton taxa and a decline in total mesozooplankton biomass in late summer through winter. While we did not find evidence of the appearance of new species, the changes in the relative abundance of *C. finmarchicus* and other copepod species resulted in a more even mesozooplankton community post-2010.

### Mesozooplankton biomass

The decline in total mesozooplankton biomass in late summer through winter was driven by the change in abundance of late-stage *C finmarchicus*, which are 5–10 times larger than most other common copepods in the Gulf of Maine (Mauchline, 1998). The increase in other zooplankton taxa, particularly *C. typicus* and *O. similis* in late summer/early autumn and Ctenophora in autumn winter (from 1.5 to 4.5% of total abundance), was not sufficient to offset the decline in late-stage *C. finmarchicus*, and there was little change in the estimated proportion of *C. finmarchicus* to the total biomass during the study period (Fig. 4). In the spring, the abundance of *C. finmarchicus*, *M. lucens*, *Pseudocalanus* spp. and *O. similis* increased significantly, but there was no concurrent increase in total zooplankton biomass. The increased abundance of *C. finmarchicus* was mainly observed in the earlier copepodid stages (Runge *et al.*, 2025), which are considerably smaller than the older stages, and therefore provides a smaller contribution to the total biomass.

**Table 4:** *Ratios showing estimated relative effects of increases in 0–50 m temperature and chlorophyll-a at the WBTS station on juvenile growth rate*, *egg production rate and mortality rates*

	Winter		Spring		Summer	
	Temperature	Chl *a*	Temperature	Chl *a*	Temperature	Chl *a*
**Broadcasters**						
Juvenile growth rate	1.13	1.41	1.11	1.16	1.19	1.27
Egg production rate	1.10	1.72	1.08	1.13	1.14	1.54
Mortality	1.01		1.07		1.11	
***Pseudocalanus***						
Juvenile growth rate	1.13	1.02	1.11	1.00	1.18	1.01
Egg production rate	1.26	1.50	1.22	1.09	1.38	1.34
Mortality	1.10		1.10		1.14	
***Oithona***						
Juvenile growth rate	1.13	1.02	1.11	1.00	1.18	1.01
Egg production rate	1.26	0.96	1.22	0.96	1.38	0.98
Mortality	1.10		1.10		1.14	

Median 0–50 m temperature (°C) from Table 3 and median chlorophyll-*a* (μg liter^−1^) in each of the three seasonal periods during 2005–2009 and 2011–2017 from Fig. 9 (standing stock divided by 50 m). Values show ratio of rate estimated for 2011–2017 over rate estimated for 2005–2009. Egg production rate (eggs female^−1^ d^−1^) as a function of temperature (under food-saturated conditions) and chlorophyll-*a* concentration for broadcast spawners (*Calanus*, *Centropages*, *Paracalanus*, *Metridia*) and for sac spawners *Pseudocalanus* and *Oithona* spp. from empirical regressions provided by Bunker and Hirst (2004): tables 4 and 6. Juvenile broadcaster and sac spawner growth rates (d^−1^) as a function of temperature (under food-saturated conditions) and chlorophyll-*a* concentration from Hirst and Bunker (2003): tables 3 and 5. Mortality rate (d^−1^) for broadcasters (DW set at 100 μg) and sac spawners (DW set at 10 μg) as a function of temperature from empirical regressions in Hirst and Kiørboe (2002)

The biomass of samples from the WBTS and CMTS were measured from preserved samples (typically 1–3 months after collection), following the Canadian AZMP protocol (Mitchell *et al.*, 2002). The possibility that some of the *Calanus* lipid stores escaped into the preservative and were not retained for biomass measurement could be a potential source of error. Nevertheless, the mesozooplankton biomass at the WBTS station, ranging on average between 10 and 30 g/m^2^ even after recent declining trends, is high compared to other areas in the North Atlantic subarctic biome, reflecting the high productivity of the western Gulf of Maine subarctic ecosystem and its aggregation in the deep basin. Peak biomass, measured as dry weight, on the Scotian Shelf reached ~6–7 g/m^2^ (Casault *et al.*, 2024), similar to the Maine Coastal Current and much lower (2–5×) than in Wilkinson Basin. In the Gulf of Saint Lawrence, historical biomass values typically range from 5 to 10 g/m^2^ and reach up to 20 g/m^2^ in the northwestern Gulf in the early summer (de Lafontaine, 1991). Biomass from the Norwegian Sea is of the same order as in the western Gulf of Maine, ranging from 10 to 20 g/m^2^ (Melle *et al.*, 2004). Data from the Barents Sea, which has also generally experienced a decrease in biomass under warming temperatures and increases in the abundances of smaller zooplankton, show a range of biomass between 5 and 10 g/m^2^ (Skjoldal *et al.*, 2022; Skjoldal, 2023).

### Mesozooplankton community composition and abundance: the seasonal interplay among advection, temperature, food availability and trophic drivers

The change in relative abundances of the mesozooplankton community associated with the oceanographic shift varied by season, determined by seasonal drivers and life cycles of the zooplankton species that play a role in the region’s ecosystem. The decline in abundance in late summer through winter of *C. finmarchicus* is likely a consequence of a decrease in net advective supply from upstream sources mediated by local summer production in the Maine Coastal Current (Runge *et al.*, 2015; Ji *et al.*, 2017; Runge *et al.*, 2025; Thompson *et al.*, 2025) and by increased predation, especially by invertebrate predators on late-stage *C. finmarchicus* in summer through winter (Wiebe *et al.*, 2022; Honda *et al.*, 2024; Honda *et al*., in review). Notably, Chaetognatha and Cnidaria, which are important predators of *C. finmarchicus*, increased in abundance at WBTS post-2010 (Fig. 7; Pershing and Kemberling, 2023; Honda *et al.*, 2025). The role of advective supply in the population dynamics of other copepod species is less certain. A comparison of the copepod assemblage in the western Gulf of Maine after 2010 with the copepod assemblage on the Scotian Shelf, which is assessed with the same methodology (Casault *et al.*, 2024), also shows an increase in the abundance of smaller copepods, coinciding with higher temperatures (Casault *et al.*, 2024). The higher proportion of *Pseudocalanus* relative to *C. typicus* in the Maine Coastal Current more closely resembles that on the Scotian Shelf, whereas *C. typicus* predominates over *Pseudocalanus* in Wilkinson Basin. In the Maine Coastal Current and on the Scotian Shelf, the abundance of *M. lucens* does not peak in the spring, as observed in Wilkinson Basin and stays consistent throughout the year (Casault *et al.*, 2024). These differences point to the Scotian Shelf as an advective source for *Pseudocalanus* spp. (likely two species: Ji *et al.*, 2009), but to the Warm Slope Water west of the Gulf Stream as a primary source for *C. typicus.* However, synchrony in trends in subpopulations in the two regions may also be due to synchrony in environmental drivers associated with local production and loss (the Moran effect: Honda *et al.*, 2023).

We hypothesize that the notable increase in spring *C. finmarchicus* (predominantly the early life stages CI–CIV: Runge *et al.*, 2025) and other copepods, as well as the summer increase in *C. typicus*, is likely a result of the increase in food availability combined with increases in ambient water temperature (Table 3; Fig. 9). A significant increase in integrated chlorophyll concentration, a proxy for phytoplankton standing stock, was observed in August through March at the WBTS station (Fig. 9). The increase in chlorophyll-*a* (chl *a*) concentrations in late summer through winter, even if at the relatively low level of 25–50 mg m^−2^ in the 0–50-m layer (equivalent to a mean concentration of 0.5–1 μg chl *a* liter^−1^) likely contributed to stronger winter/spring reproduction of *C. finmarchicus* (Runge *et al.*, 2006, 2025), perhaps combined with shorter duration of diapause driven by warmer temperatures (Maps *et al.*, 2012). Examples of the effect of early food availability on *C. finmarchicus* reproduction in the western Gulf of Maine are the high abundance of nauplii and early copepodid stages in late February 1999, when mean surface layer (0–40 m) chlorophyll-*a* concentrations ranged between 0.5 and 3.5 μg liter^−1^, compared to low abundance at the same time in 2000, when mean chlorophyll-*a* concentrations were < 0.5 μg liter^−1^ (Durbin *et al.*, 2003). Similarly, a large autumn bloom occurred in Wilkinson Basin in autumn 2012 and February–March 2013, when chlorophyll-*a* concentrations were > 0.5 μg liter^−1^ (much of it subsurface), leading to an early and unusually high abundance (252, 578 ind. m^−2^) of stage CI–CIV by early May 2013 (Runge *et al.*, 2015).

The higher autumn and late winter phytoplankton standing stocks and increased ambient water temperatures likely supported the egg production and growth rates of other copepod species. *Metridia lucens*, a relatively larger, broadcast-spawning copepod, appears to be quiescent in late autumn and winter, and reproduces in response to the spring bloom (Durbin *et al.*, 2003). Its abundance in Wilkinson Basin after 2010 peaks in the spring and is lower from the summer through the winter. *Centropages typicus*, a more warm water–adapted broadcast spawner (Ji *et al.*, 2009), which Durbin and Kane (2007) concluded is historically food limited in late autumn and winter in the Gulf of Maine, could also have been the beneficiary of higher summer through winter chlorophyll standing stocks to expand its population abundance. *Pseudocalanus* spp. carry their eggs until hatching, develop relatively rapidly in colder water and recruit in late winter through early summer, after which the population is likely controlled by predation (Davis, 1984; Ji *et al.*, 2009). *Oithona similis*, a very small, ubiquitous species in the North Atlantic (Bigelow, 1924), became dominant in abundance through most of the year (except spring) after 2010.

We did not find a significant difference in mixed layer depth before and after 2010 in any season (Table 3). Since the critical depth is much less than the observed mixed layer depth in winter, the particularly critical season for increased egg production rates, it is likely that increased chlorophyll-*a* in winter was not due to a change in stratification. Durbin *et al*. (2003) suggested that a shallow surface layer of fresher Scotian Shelf water may have been conducive to support early phytoplankton growth, although it seems unlikely that this would be a persistent feature over successive years. Contrary to the prevailing theory that stratification drives the initiation of the spring bloom in temperate and sub-Arctic latitudes (Sverdrup, 1956), a growing number of observations, including from the GoM, show that phytoplankton growth in the spring begins before stratification is established (Townsend *et al.*, 1992, Behrenfeld and Boss, 2018). It has been proposed that the bloom dynamics is governed by processes that decouple phytoplankton from their grazers (Behrenfeld, 2010). We further suggest that the decline of late-stage *C. finmarchicus* emerging from diapause and feeding in late winter/early spring also played a role in favoring phytoplankton growth rate over mortality loss, both through a reduction in grazing pressure and also a reduction in predation on eggs and nauplii of other copepod species (Pershing and Kemberling, 2023; Runge *et al.*, 2025). Hence, the shift in relative abundances of mesozooplankton before and after 2010 likely involves a cascade of top-down (invertebrate predators–lower *C. finmarchius* abundance–lower phytoplankton grazing rates) and bottom-up (higher food availability–higher autumn/winter copepod production) effects (Pershing and Kemberling, 2023: Honda *et al.*, 2024; Honda et al., 2025).

### What are the relative effects of temperature and food availability on copepod production before and after 2010?

In Table 4, we applied global empirical relationships from the literature to explore the relative effect of increases in temperature and food availability on copepod growth, egg production and mortality rates in the periods before and after 2010. Temperature averaged over the upper 50 m is assumed to represent the copepod’s ambient environment. Chlorophyll-*a* concentration averaged over the upper 50 m as a proxy for omnivorous copepod food availability is supported by previously observed relationships with copepod egg production rate (e.g. Runge, 1985; Runge *et al.*, 2006). The predicted increases in egg production due to increases in median chlorophyll-*a* after 2010 were 54–72% in late summer through winter for broadcast spawners, compared to 10–14% due to increases in ambient temperature (Table 4). For egg-carrying species (represented here by *Pseudocalanus* spp.), the predicted increase in egg production due to increases in chlorophyll-*a* were 34–50% compared to 26–38% due to higher ambient temperature. These differences are due to the sensitivity of the egg production–chlorophyll relationship in the range of 0.5–2 μg/liter chlorophyll-*a* (e.g. Runge *et al.*, 2006) compared to relatively small increases expected with a < 2°C rise in ambient temperature (Bunker and Hirst, 2004). The observed increases in chlorophyll are predicted to have more effect than temperature on juvenile growth rate of broadcast spawners as well, although, interestingly, increases in temperature have more effect than chlorophyll increases on juvenile growth rates of egg-carrying species (Table 4).

The effects of increases in food availability on egg production rate would be particularly evident for *Centropages* in autumn through winter and *C. finmarchicus* in late winter and spring (Table 4; Fig. 5; Runge *et al.*, 2025). In contrast, temperature increase had a more dominant effect than chlorophyll-*a* concentration on egg production rate of *O. similis*, which feeds on ciliates, flagellates and likely nauplii of small copepod species and fecal pellets (Castellani *et al.*, 2005). For both broadcast and egg sac spawners, the combined effects of temperature and chlorophyll-*a* increase were greater than the predicted increase in mortality rate due to temperature (Table 4). While the empirical mortality relationship may not have adequately reflected increases in predators after 2010 (Fig. 7; Pershing and Kemberling, 2023), the analysis nevertheless supports a primary role of food availability rather than temperature increase in driving increased productivity of the smaller copepods, as well as *C. finmarchicus* in spring (Runge *et al.*, 2025) after 2010.

## CONCLUSION

The data from the WBTS station show a shift in the mesozooplankton community between the ~5-year periods before and after the oceanographic shift in 2010. The shift did not involve a change in species composition, but rather a substantial decline in mesozooplankton biomass between August and March, driven by declines in late-stage abundance of *C. finmarchicus*. The increases in abundance of most other mesozooplankton taxa after 2010 were not sufficient to offset this decline in biomass. While the duration of the CMTS station was too short to make a robust comparison before and after 2010, the copepod species composition was similar to the WBTS station, but with enhanced contribution of more coastal species such as *Pseudocalanus* spp. and *Temora longicornis*. The productive Maine Coastal Current, which the CMTS station represents, is considered to be an important local source of supply of *C. finmarchicus* to Wilkinson Basin and southern New England (Runge *et al.*, 2015, 2025). However, the low abundance of potential invertebrate predators (e.g. Ctenophores and Chaetognaths) at the CMTS station suggests that the coastal current is not a source of predators to the deeper Wilkinson Basin, which must sustain its invertebrate predator population by local production and/or advection from other, deeper sources.

Ecologically significant increases in chlorophyll-*a* standing stock in summer and winter combined with increased water column temperatures in the mixed layer in fall and winter are consistent with the hypothesis that these environmental variables drove the increases in young-stage *C. finmarchicus* in spring and other planktonic copepods throughout the year. Building on the analysis by Pershing and Kemberling (2023), we hypothesize that late-stage *C. finmarchicus*, in addition to serving as a lipid-rich nutritional foundation for higher trophic levels in the subarctic Gulf of Maine ecosystem, also play a role as a competitive dominant, especially during late summer through winter. When late-stage *C. finmarchicus* abundance is high, food availability to other grazers is suppressed, and predation by *C. finmarchicus*, including eggs of smaller, broadcast-spawning copepods, is higher. When late-stage abundance is low, the balance between phytoplankton growth and loss tips more to growth, resulting in more food availability. Shifts from low to high late-stage abundance of *C. finmarchicus* are hypothesized to be driven by shifts in external supply from the western Scotian Shelf and reduced predation (Ji *et al.*, 2021; Honda *et al.*, 2025; Runge *et al.*, 2025; Thompson *et al.*, 2025).

The possibility of an ecological, predator–prey cycle, suggested by Pershing and Kemberling (2023) and Honda *et al*. (2024, in review), in which late summer through winter predation suppresses late-stage *C. finmarchicus* abundance, which in turn releases more summer and late winter/early spring phytoplankton standing stock as well as reduced predation by *C. finmarchicus* on early life stages of other copepod species, merits further consideration. We suggest that this cycle may be interrupted at decadal level scales by large immigration events of *C. finmarchicus* from the western Scotian Shelf, driven by shifts in basin-scale circulation (e.g. Saba *et al*., 2016; Gonçalves Neto *et al*. 2021; Seidov *et al.*, 2021) and water mass transport into the Gulf of Maine (e.g. Meyer-Gutbrod *et al*., 2021; Townsend *et al.*, 2023). Whether this pattern would continue under relentless atmospheric CO_2_ increase (Ripple *et al.*, 2024) remains to be seen.

Our hypotheses and conclusions here and elsewhere (Honda *et al.*, 2025; Runge *et al.*, 2025) support alternative interpretations to statistical analyses reported by Nowakowski *et al.* (2025), which indicate that warming temperature has overtaken stratification (mixed layer depth) as the primary driver of change in copepod abundance in the Gulf of Maine. A combination of supply from Scotian Shelf water, predation and food availability in late summer through winter, varying in seasonal through multiyear time scales, offers a coherent explanation of copepod abundance results. If surface warming has emerged as a primary driver, we suggest it is a proxy for change in these underlying mechanisms.

Although the post-2010 oceanographic shift has been called a regime shift, the subarctic community structure of the mesozooplankton did not change. Many of the variables we measured are adequately described by a linear regression (Figs. S1–S3) rather than a step shift from one value to another. The truly radical regime shift (sensu Bakun, 2004) may come when the synergistic processes controlling food availability, local production and advective supply of *C. finmarchicus* breaks down with relentless warming, and this key species, along with the subarctic ecosystem it supports, is lost from the Gulf of Maine. It is clear that continued, timely time-series observations of planktonic communities in the Gulf of Maine are essential for understanding change in structure and function of the region’s ecosystem.

## DATA AVAILIBILITY

The biomass and *Calanus finmarchicus* abundance data presented in this study are available on ERDAPP via the following links:

WBTS CTD data:

https://data.neracoos.org/erddap/tabledap/WBTS_CTD.html

WBTS biomass and *Calanus finmarchicus* abundance 2004–2017:

https://data.neracoos.org/erddap/tabledap/WBTS_CFIN_2004_2017.html

CMTS biomass and *Calanus finmarchicus* abundance 2007–2017:

https://data.neracoos.org/erddap/tabledap/CMTS_CFIN_2007_2017.html

## Supplementary Material

Supplementary_materials_fbaf066
