# Supplementary material for: Response of the mesozooplankton community in the western Gulf of Maine to changing oceanographic conditions: the 2010 regime shift
Source: J Plankton Res. 2026 Jan 7;48(1):fbaf066. doi: 10.1093/plankt/fbaf066 (PMC12777979; doi:10.1093/plankt/fbaf066)
Supplement: Supplementary_materials_fbaf066 [file supplementary_materials_fbaf066.docx]

**SUPPLEMENTARY FIGURES**


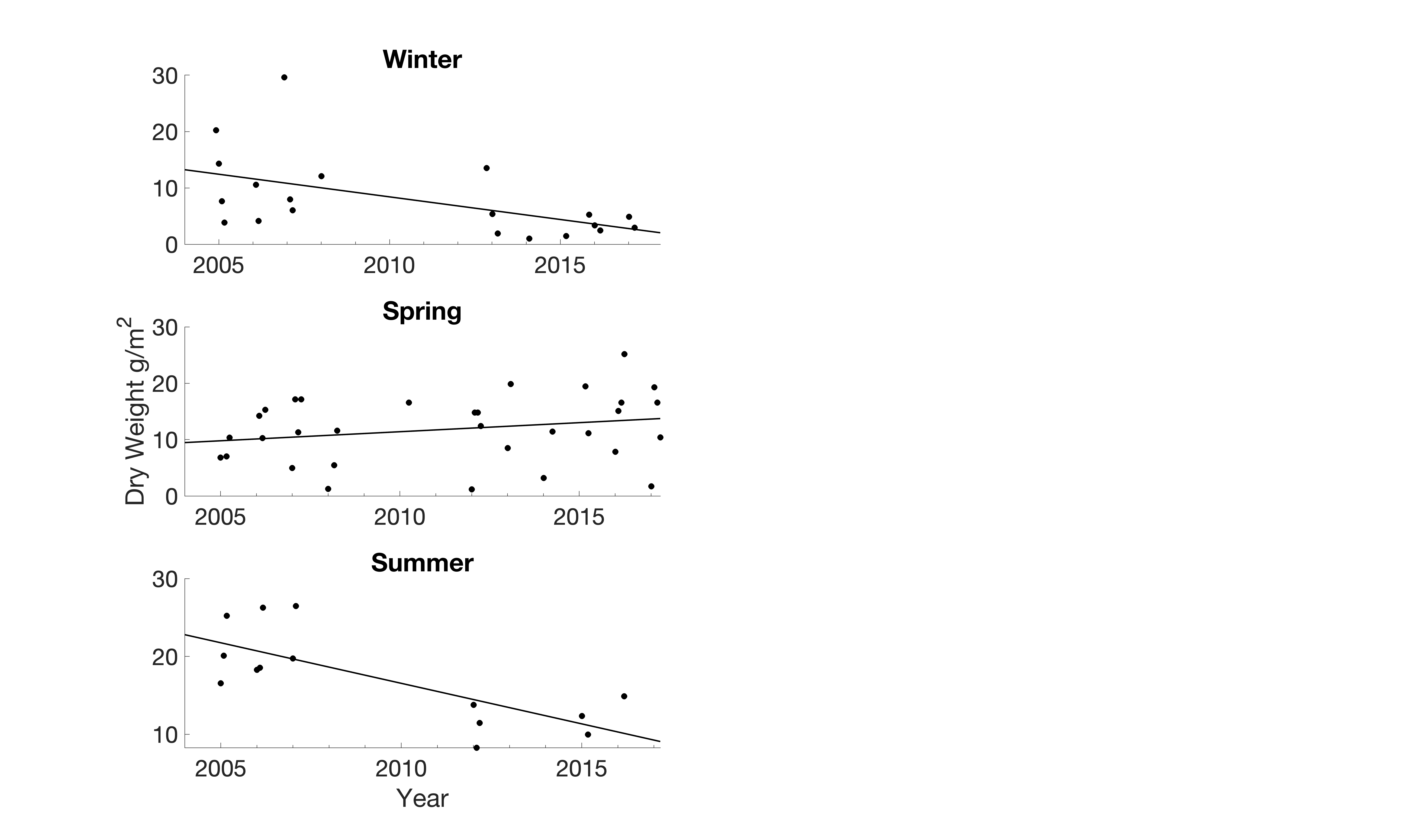

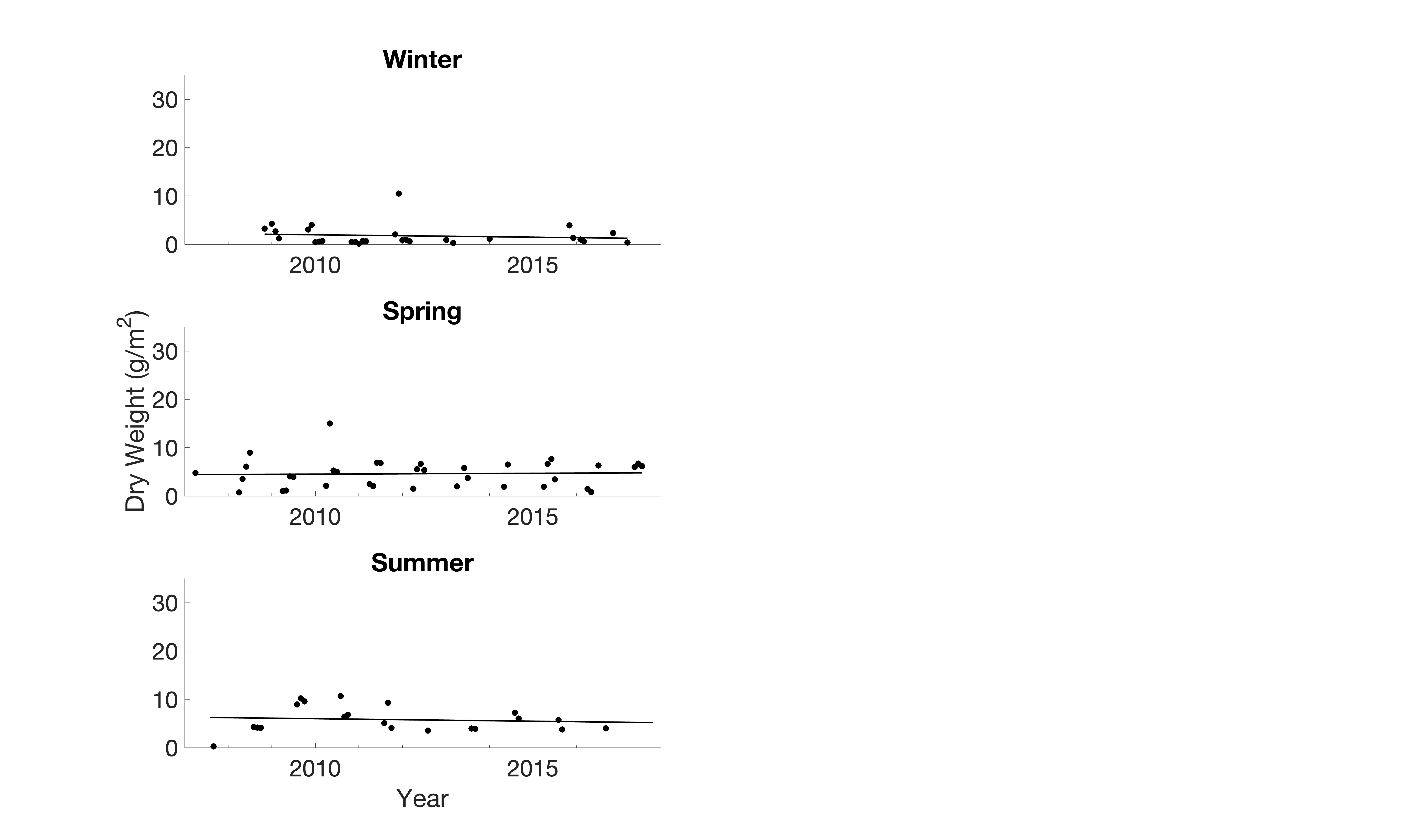


**Figure S1**. Mesozooplankton dry weight (g/m^2^) at the Wilkinson Basin Time Series station (left panel) and Coastal Maine Time Series station (right panel) between 2005 and 2017. At WBTS, biomass significantly decreased in the winter (y = -0.002x + 13.228, p = 0.016, r^2^ = 0.284) and the summer (y = -0.003x + 22.797, p = 0.002, r^2^ = 0.553, and no significant trend in the spring (y = 0.001x + 11.390, p = 0.199, r^2^ = 0.054). At CMTS there were no significant trends in the winter (y = -0.000x + 2.264 , p = 0.536, r^2^ = 0.015), spring (y = -0.000x + 4.420, p = 0.830, r^2^ = 0.001), or summer (y = -0.000x + 6.251, p = 0.658, r^2^ = 0.011). In the regression analysis x = yearday since Jan 1^st^, 2004 at WBTS and Jan 1^st^, 2007 at CMTS.


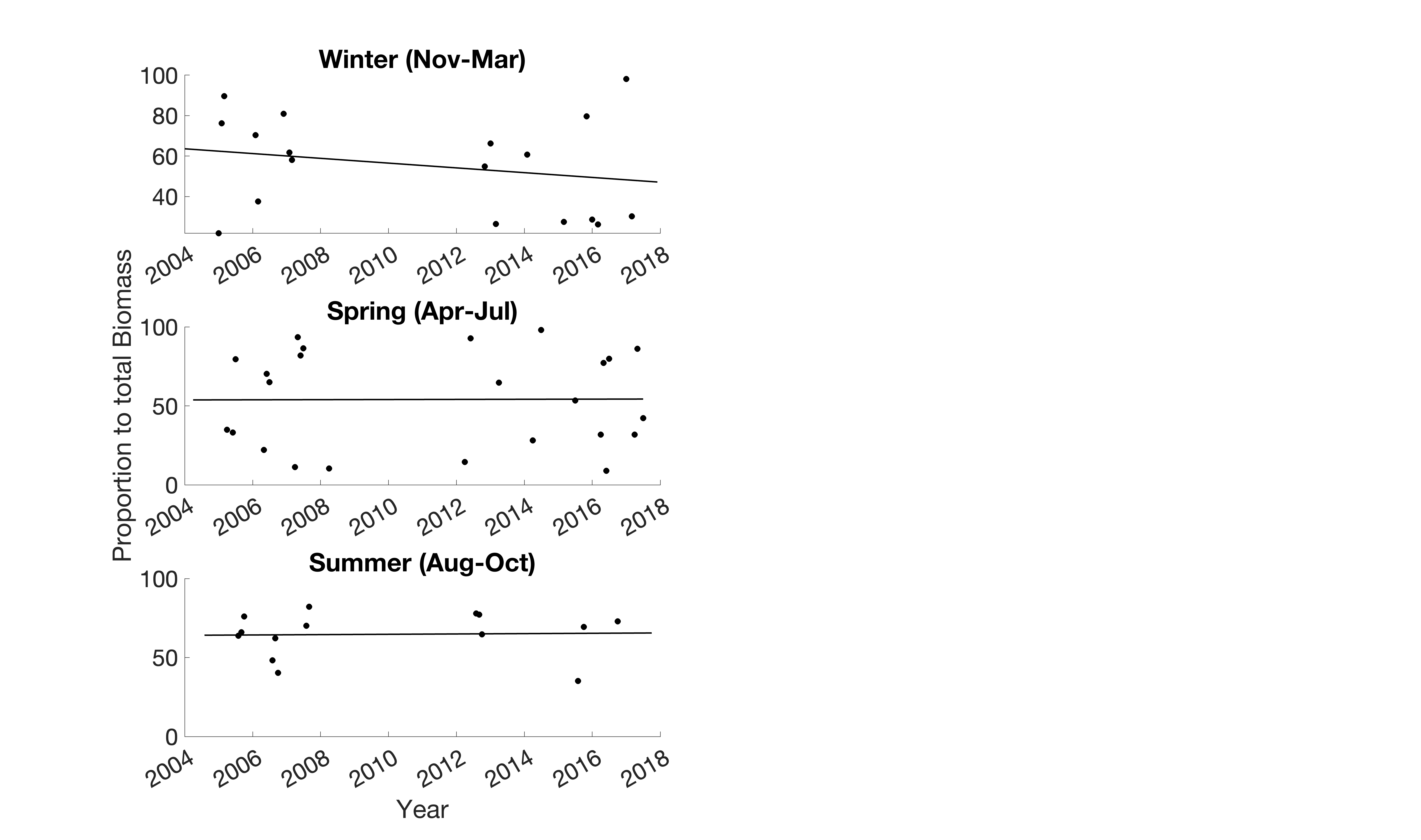


**Figure S2**. Estimated contribution (%) of *Calanus finmarchicus* (stages CI-CV) biomass, as determined by published dry weight values, to the total zooplankton biomass at the Wilkinson Basin Time Series station (WBTS) between 2005 and 2017. Winter: y = -0.003x + 63.614 ( *p = 0.362*, r^2^ = 0.052); spring: y = 0.000x + 53.759 (*p = 0.977*, r^2^ = 0.000); summer: y = 0.001x + 64.146 (*p = 0.914*, r^2^ = 0.001), where x = yearday since Jan 1^st^, 2004.


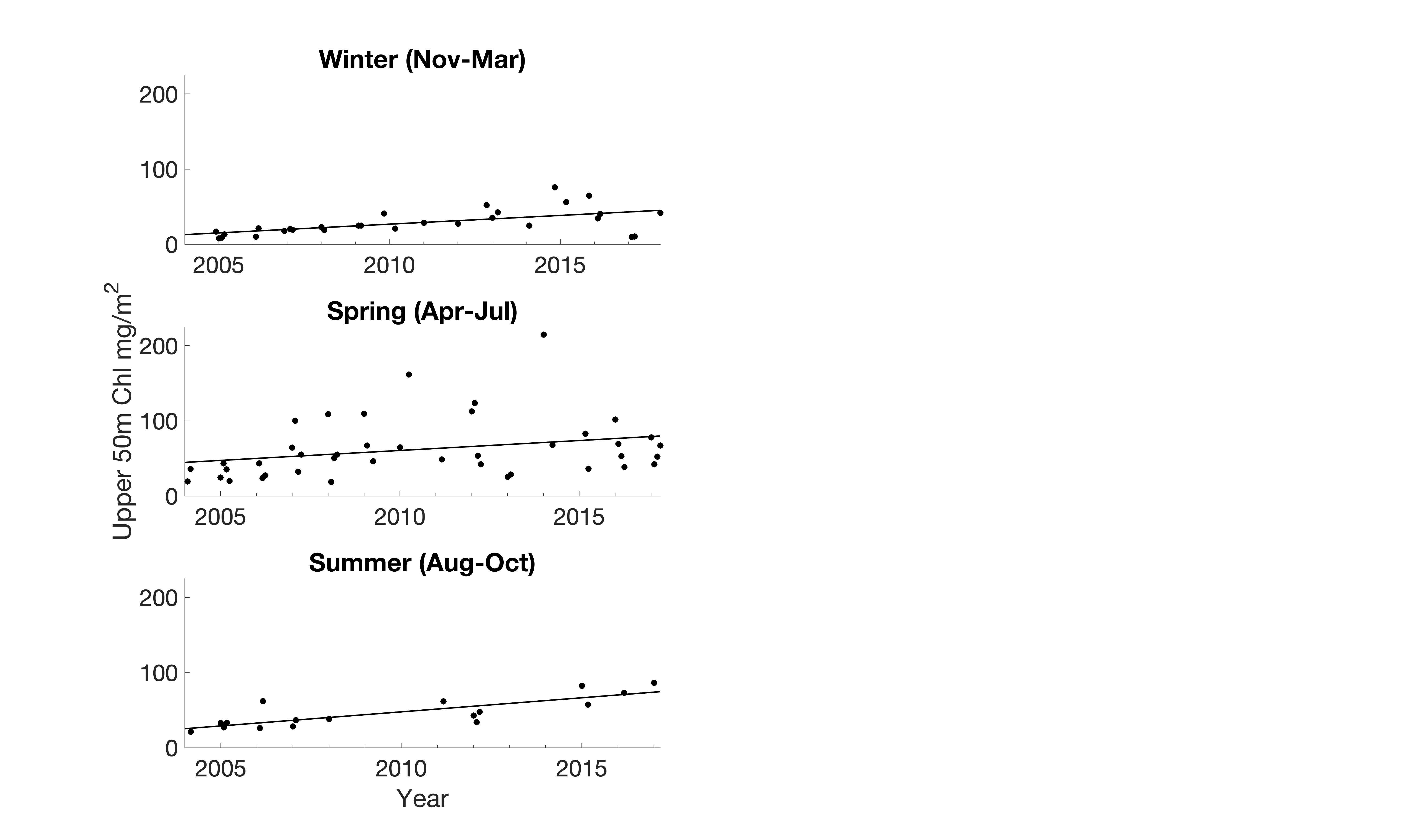


**Figure S3**. Time series of integrated chlorophyll a concentration (mg Chl a m^-2^) in the upper 50m of the water column at the Wilkinson Basin Time Series station during the winter, spring and summer. Chlorophyll a standing stock significantly increased in the winter (y = 0.006x + 12.835, p = 0.001, r^2^ = 0.332) and summer (y = 0.010x + 25.205, p = 0.000, r^2^ = 0.656) and no significant trend in the spring (y = 0.007x + 44.671, p = 0.078, r^2^ = 0.077). In the regression analysis x = yearday since Jan 1^st^, 2004.
